# Targeted Disruption of *TgPhIL1* in *Toxoplasma gondii* Results in Altered Parasite Morphology and Fitness

**DOI:** 10.1371/journal.pone.0023977

**Published:** 2011-08-25

**Authors:** Whittney Dotzler Barkhuff, Stacey D. Gilk, Ryan Whitmarsh, Lucas D. Tilley, Chris Hunter, Gary E. Ward

**Affiliations:** 1 Department of Microbiology and Molecular Genetics, University of Vermont, Burlington, Vermont, United States of America; 2 Department of Pathobiology, University of Pennsylvania, Philadelphia, Pennsylvania, United States of America; Université Pierre et Marie Curie, France

## Abstract

The inner membrane complex (IMC), a series of flattened vesicles at the periphery of apicomplexan parasites, is thought to be important for parasite shape, motility and replication, but few of the IMC proteins that function in these processes have been identified. TgPhIL1, a *Toxoplasma gondii* protein that was previously identified through photosensitized labeling with 5-[^125^I] iodonapthaline-1-azide, associates with the IMC and/or underlying cytoskeleton and is concentrated at the apical end of the parasite. Orthologs of TgPhIL1 are found in other apicomplexans, but the function of this conserved protein family is unknown. As a first step towards determining the function of TgPhIL1 and its orthologs, we generated a *T. gondii* parasite line in which the single copy of *TgPhIL1* was disrupted by homologous recombination. The *TgPhIL1* knockout parasites have a distinctly different morphology than wild-type parasites, and normal shape is restored in the knockout background after complementation with the wild-type allele. The knockout parasites are outcompeted in culture by parasites expressing functional *TgPhIL1,* and they generate a reduced parasite load in the spleen and liver of infected mice. These findings demonstrate a role for TgPhIL1 in the morphology, growth and fitness of *T. gondii* tachyzoites.

## Introduction

The Phylum Apicomplexa contains a number of medically important parasites including *Cryptosporidium spp.*, which cause diarrheal illness in children and immunocompromised patients, *Plasmodium spp.*, which are the causative agents of malaria, and *Toxoplasma gondii*, which causes life-threatening disease in immunocompromised people and the developing fetus. Other apicomplexan organisms, including *Neospora caninum* and *Eimeria spp.*, cause severe disease in domestic animals. Despite the significant morbidity, mortality and economic loss caused by these pathogens, information is lacking regarding many aspects of their basic biology. *T. gondii* is a powerful model system for studying conserved aspects of apicomplexan biology, due to the ease with which *T. gondii* can be cultured and genetically manipulated [1], [2].

Apicomplexan parasites contain a number of unusual subcellular structures and organelles [3], [4], including the inner membrane complex (IMC), a series of flattened vesicles that are tightly apposed to the cytosolic face of the plasma membrane [5], [6]. Together, the plasma membrane and IMC are referred to as the pellicle. Underlying the IMC is the subpellicular network, a meshwork of intermediate filament-like proteins [3], [7], [8]. Additional cytoskeletal structures associated with the pellicle include the conoid, which is a cone-shaped structure composed of a novel polymeric form of tubulin [9], a pair of polar rings located at either end of the conoid [9], [10], and 22 microtubules radiating posteriorly from the lower polar ring [10].

Various functions for the IMC, subpellicular network, and subpellicular microtubules have been proposed [3], [4], [8], [11]. During parasite replication, a process known as endodyogeny, the IMC forms early and may provide a scaffold upon which daughter parasites are assembled, as well as mechanical stability for the mature tachyzoites [3], [7], [12]. Additionally, some components of the myosin motor complex, the proteins that provide the mechanochemical force for parasite motility and host cell invasion, are anchored in the IMC. Localization of the motor complex between the outer membrane of the IMC and the plasma membrane is thought to be critical for motility and invasion [13]–[15]. While one member of the myosin motor complex, GAP45, appears to physically connect the IMC to the plasma membrane [16], and the recently identified GAPM proteins may function to anchor the subpellicular network to the flattened IMC vesicles [17], the detailed mechanism(s) by which the IMC, subpellicular network and subpellicular microtubules associate with each other and are organized at the parasite periphery have yet to be elucidated.

TgPhIL1 (*Ph*otosensitized *I*NA-*L*abeled protein 1) was previously identified through photosensitized labeling with 5-[^125^I] iodonapthaline-1-azide (INA) and localized to the parasite periphery, concentrated at the apical end directly posterior to the conoid [18]. TgPhIL1 is highly conserved among members of the Phylum Apicomplexa but shows little homology to proteins outside of the phylum. When parasites were treated with *Clostridium septicum* alpha-toxin, which causes the plasma membrane to swell away from the IMC [19], TgPhIL1 remained associated with the IMC and/or associated cytoskeletal structures [18]. Although TgPhIL1 labeling with INA suggests this protein is membrane associated, TgPhIL1 is also highly insoluble and extracts much like a cytoskeletal protein [18].

In order to learn more about the function of TgPhIL1, we generated parasites containing a *TgPhIL1* deletion. Phenotypic analysis of the knockout parasites revealed that TgPhIL1 is necessary for maintaining the distinctive shape of *T. gondii* tachyzoites, and parasites lacking TgPhIL1 show both a growth defect in culture and reduced fitness in a mouse model of infection.

## Materials and Methods

### Ethics statement

All mouse experiments were performed with approval of the Institutional Animal Care and Use Committee (IACUC) of the University of Pennsylvania (Protocol # 801490; "Regulation of the early response to *Toxoplasma gondii*").

### Culture of parasites

Wild-type RH strain *T. gondii* was maintained by serial passage in confluent primary human foreskin fibroblast (HFF) cells in Dulbecco's Modified Eagle's Medium (DMEM) supplemented with 1% (vol/vol) heat inactivated fetal bovine serum (FBS), 10 units/ml penicillin G, 10 µg/ml streptomycin sulfate, and 10 mM HEPES buffer, as previously described [20].

### Generation of the *TgPhIL1* knockout parasite line (RH*ΔTgPhIL1)*

All PCR reactions were performed with Pfx polymerase (Invitrogen, Carlsbad CA) unless otherwise indicated. All primers were synthesized by Sigma Genosys (The Woodlands TX) and restriction enzymes were purchased from either New England BioLabs (Ipswich MA) or Invitrogen.

In order to generate the *TgPhIL1* knockout construct, PCR was performed on *T. gondii* genomic DNA using primers PhIL15′KO_for_ (5′GCTGAGGAGGGGAAGAAGATCGAG3′) and PhIL13′KO_rev_ *Xba*I (5′TGCTCTAGATATCCGCAAATGCTCGCTCGCC3′) to generate a 6.5 kb amplicon containing 2.9 kb of *TgPhIL1* 5′ flanking sequence, the 1.3 kb *TgPhIL1* open reading frame (ORF), and 3.1 kb of *TgPhIL1* 3′ flanking sequence. The amplified product was cloned into pCR-Blunt TOPO (Invitrogen) and inverse PCR was performed with primers KOinv_for_ (5′AGGAGTGTTCATGTTGTTTGCTG3′) and KOinv_rev_ (5′TGAATTCGTCTAATCCAGAGTCTGTC3′) to remove the *TgPhIL1* ORF. The *ble* cassette was digested from p*GRA1/ble* [21] with *Hind*III and *Xba*I and the ends were filled in with Klenow, followed by blunt ligation to the inverse PCR product in order to generate the knockout construct pΔ*PhIL1/ble*. The plasmid was linearized with *Not*1 and transfected into RH parasites. The transfected parasites were added to confluent HFF cells and selected with phleomycin as described previously [21]. Individual clones were isolated by limiting dilution and clones lacking *TgPhIL1* expression were identified by immunofluorescence with anti-TgPhIL1 [18].

### Cloning *TgPhIL1* under its endogenous promoter for complementation

PCR was performed on *T. gondii* genomic DNA using the primers PhIL-Endo_for_ -*Xba*I (5′GGGGTCTAGATGAAAGACTGGAGCATTTCG-3′), corresponding to sequence 2 kb upstream of the *TgPhIL1* ORF, and PhIL1-Endo_rev_-*Bgl*II (5′GGGGAGATCTTCACCGAGAGAAGTCGAGTG-3′) containing the 3′ end of the *TgPhIL1* ORF, followed by a stop codon. After digestion with *Xba*I and *Bgl*II, the product was ligated into p*tubIMC1-YFP/sagCAT* [22] which had also been digested with *Xba*I and *Bgl*II in order to remove the tubulin promoter and *TgIMC1* ORF, generating p*TgPhIL1Endo/sagCAT*. Complemented parasites were maintained in 20 µM chloramphenicol.

### Western blot analysis

Western blot analysis of parasite lysates was performed as described previously [23], using either a rabbit polyclonal anti-TgActin antibody generously provided by Dr. David Sibley [24] or rabbit polyclonal anti-TgPhIL1 [18], at dilutions of 1∶10,000 and 1∶5,000 respectively.

### Immunofluorescence

Parasites were attached to glass coverslips with BD-CellTak (BD Biosciences, San Jose CA) as previously described [23], fixed in phosphate-buffered saline (PBS) containing 4% (vol/vol) paraformaldehyde for 10 minutes at 23°C and permeabilized for 30 minutes at 23°C in PBS containing either 10 mM deoxycholic acid (for anti-α-tubulin and anti-TgPhIL1) or 0.25% (vol/vol) Triton X-100 (for anti-ISP1 and anti-*T. gondii* polyclonal serum). The permeabilized parasites were incubated for 15 minutes in PBS containing 2% (wt/vol) BSA (PBS-2% BSA) and one or more of the following primary antibodies: anti-TgPhIL1, diluted 1∶500; mouse monoclonal anti-α-tubulin (Sigma-Aldrich, St. Louis MO), diluted 1∶400; mouse monoclonal anti-ISP1 (monoclonal 7E8; [25]), diluted 1∶2000; and rabbit polyclonal anti-*T. gondii* (catalog #90700556; AbD Serotec, Raleigh, NC), diluted 1∶2000. Immunofluorescence with anti-TgMORN1 ([26]; 1∶500), and anti-TgIMC1 ([23], 1∶100) was performed after fixation of infected HFF monolayers on glass coverslips in 100% MeOH on ice for 5 minutes. After incubation with primary antibodies, the samples were incubated with a 1∶1000 dilution of Alexa488- and/or Alexa546-conjugated secondary antibody (Invitrogen).

### Morphometric analysis

Parasites resuspended in Hanks Buffered Salt Solution (HBSS; Invitrogen) containing 10 mM HEPES, pH 7.2, were attached to either glass coverslips or 8-well chambered coverglasses (Nunc, Rochester NY) using BD-CellTak, incubated for 15 minutes at 23°C with or without various concentrations of mercuric chloride (HgCl_2_), and visualized by differential interference contrast (DIC) microscopy. The widest point along both the long and short axes of the parasite was determined using ImageJ (http://rsbweb.nih.gov/ij/).

### Electron microscopy

Parasites were pelleted at 1,000 x*g*, resuspended in Karnovsky's reagent (1% [wt/vol] paraformaldehyde, 2.5% [vol/vol] glutaraldehyde) and incubated for 60 minutes at 4°C. The fixed cells were rinsed 3 times (4 minutes each) in Millonig's phosphate buffer [27] and embedded in 2% (wt/vol) SeaPrep Agarose (Cambrex BioScience Rockland, Rockland ME), for 15 minutes at 4°C. The samples were then incubated in Karnovsky's reagent for 15 minutes at 4°C, followed by washing in Millonig's buffer and trimming of the agarose blocks. 1% (wt/vol) osmium tetraoxide (OsO_4_) was added and the blocks were stored in Millonig's buffer overnight. The following day, the blocks were sequentially dehydrated in 35%, 50%, 70%, 85%, 95%, and 100% (vol/vol) ethanol. They were further dehydrated in propylene oxide, infiltrated with and embedded in Spurr's resin, and allowed to polymerize for 12 hours. Ultrathin sections were cut, placed on nickel grids and contrasted with 2% (wt/vol) uranyl acetate in 50% (vol/vol) ethanol for 6 minutes, followed by lead citrate for 4 minutes, before being analyzed on a JEOL 1210 transmission electron microscope (JEOL, Peabody MA).

### Growth competition

Freshly egressed wild-type RH, RH*ΔTgPhIL1*, and *TgPhIL1* complemented clones (RH*ΔTgPhIL1/TgPhIL1-C5* and RH*ΔTgPhIL1/TgPhIL1-C7*) were filtered through a 3-µm Nuclepore syringe filter (Whatman, Piscataway NJ). 5×10^5^ parasites of each strain were added pairwise to a 25 cm^2^ flask of confluent HFF cells, in the combinations indicated, for a total of 1×10^6^ parasites per experiment. Immediately following the lysis of the monolayers (approximately 36 hours post-infection), 0.3 ml of parasite-containing culture supernatant was added to a fresh monolayer. After every third such passage, immunofluorescence was performed on the released parasites with rabbit polyclonal anti-TgPhIL1. A total of 500 parasites/sample were scored as either negative or positive for TgPhIL1. The medium was replaced 12 hours after each infection to ensure that parasites that failed to invade were not counted in the analysis.

### Mouse infections and analysis

Female 6 to 8 week old C57BL/6 mice were obtained from Jackson Laboratories (Bar Harbor, ME) and maintained under specific-pathogen-free conditions in accordance with institutional guidelines. 5 mice per group were infected intraperitoneally with 200 µl of PBS containing 1×10^4^ RH or RH*ΔTgPhIL1* tachyzoites. Peritoneal cells were harvested on day 7 by lavage with cold PBS, spun onto coverslips and stained using the HEMA-3 kit (Fisher Scientific) as directed by the manufacturer. Parasite DNA levels were measured by real time PCR on DNA isolated from liver and spleen samples 7 days post-infection using the High Pure PCR Template Purification Kit (Roche, Indianapolis IN). The tandemly arrayed, 35-fold-repetitive *T. gondii* B1 gene [28] was amplified by real-time PCR (Forward 5′- TCCCCTCTGCTGGCGAAAAGT-3′; Reverse 5′- AGCGTTCGTGGTCAACTATCGATTG-3′) using the AB7500 fast real-time PCR thermal cycler and Power SYBR green reagents (Applied Biosystems, Foster City CA). Quantitect primers for the mouse housekeeping gene β-actin (Qiagen, Valencia CA) were used as a normalization control. The cycle numbers were normalized relative to their individual actin signals and these were averaged among the 5 mice per group; RH*ΔTgPhIL1* was set to 1.

### Laser Scanning Cytometry (LSC)-based invasion assay

Syringe-released parasites were filtered through a Nuclepore filter, washed in HBSS by centrifugation (4 minutes, 1,000 x*g*) and resuspended at a concentration of 1×10^6^ parasites/ml in HBSS containing 1% FBS. Parasites were added to confluent monolayers of HFF cells grown on 25 mm^2^ round glass coverslips in 6-well plates (3×10^6^ parasites added/well). The coverslips were incubated for 1 hour at 37°C, and processed and analyzed by LSC as described previously [29].

### Motility assay

Freshly egressed and filtered parasites were harvested, centrifuged at 1,000 x*g* for 4 minutes, and resuspended in HBSS at a concentration of 1.5×10^7^ parasites/ml. 100 µl of the parasite suspension was added to BD-CellTak-coated 8-well chambered coverglasses (Nunc, Rochester NY) and incubated at 37°C for 45 minutes. The buffer was removed by gentle aspiration, and the samples were fixed in 2.5% (vol/vol) paraformaldehyde for 15 minutes at 23°C, then blocked with PBS-2% BSA for 15 minutes. Trails were stained with 5 µg/ml mouse monoclonal anti-TgSAG1 (Argene, North Massapequa NY) in PBS-0.5% BSA for 15 minutes, washed three times in PBS, incubated for 15 minutes with Alexa 488-conjugated goat anti-mouse antibody, washed again and visualized by fluorescence microscopy.

### Conoid extension assay

Conoid extension assays were performed as described previously [30]. Briefly, freshly egressed parasites were incubated for 5 minutes at 23°C in HBSS containing 1 µM ionomycin (Sigma) or an equivalent volume of DMSO, fixed for 1 hour at 23°C in 1% (vol/vol) glutaraldehyde, smeared onto a glass slide, and air-dried. 100 conoids per sample were scored as either extended or retracted by phase contrast microscopy.

## Results

### Generation of a *TgPhIL1* knockout parasite line

**Figure 1:**
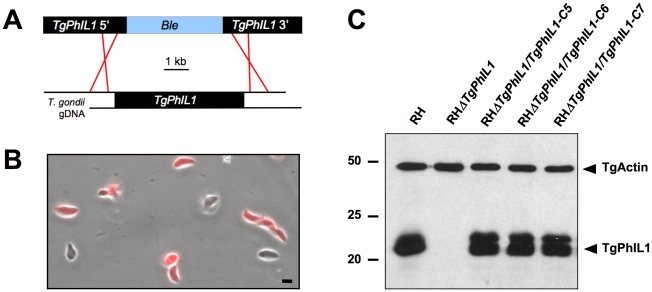
Generation of a *TgPhIL1* knockout parasite line. (**A**) A knockout construct containing a *ble* cassette, flanked by 3 kb of upstream and downstream noncoding sequence from the *TgPhIL1* genomic locus was linearized and transfected into wild-type RH parasites, where it was predicted to integrate into the *TgPhIL1* locus by homologous recombination. (**B**) After three rounds of selection with phleomycin, immunofluorescence with anti-TgPhIL1 antiserum (red) revealed that 6% of the parasites were negative for TgPhIL1, indicating successful disruption of the open reading frame. A combined phase contrast and immunofluorescence image is shown. Scale bar  = 5 µm. (**C**) Western blotting with anti-TgPhIL1 confirmed the absence of TgPhIL1 expression in the knockout parasites (RH*ΔTgPhIL1)* and restoration of TgPhIL1 expression to approximately wild-type (RH) levels in the complemented clones (RH*ΔTgPhIL1/PhIL1-C5, C6, C7*). The blot was simultaneously probed with anti-actin as a loading control. As seen here, TgPhIL1 sometimes resolves by SDS-PAGE as a tightly spaced doublet, possibly reflecting the presence of an as yet uncharacterized posttranslational modification. Numbers on the left indicate molecular mass in kDa.

*TgPhIL1* was targeted for disruption by a construct encoding *ble*, which confers resistance to the drug phleomycin [21], flanked by 5′ and 3′ sequences from the *TgPhIL1* genomic locus (Fig. 1A). Following transfection and three rounds of selection, 6% of the population lacked TgPhIL1 staining by immunofluorescence (Fig. 1B). Parasites in this population were cloned by limiting dilution, and individual clones lacking TgPhIL1 were identified by immunofluorescence. Immunoblotting with anti-TgPhIL1 antibody confirmed the lack of TgPhIL1 expression in several of the clones, one of which (RH*ΔTgPhIL1*; Fig. 1C) was chosen arbitrarily for all subsequent experiments.

For complementation studies, we reintroduced *TgPhIL1* into the RH*ΔTgPhIL1* parasites using a construct containing 2 kb of *TgPhIL1* upstream genomic sequence, the *TgPhIL1* ORF, and a chloramphenicol acetyltransferase cassette for selection. The construct was transfected into RH*ΔTgPhIL1* parasites, which were then subjected to three rounds of selection with chloramphenicol. Immunofluorescence with anti-TgPhIL1 antibody showed that stable transfectants had been generated and that the expressed TgPhIL1 localizes properly (data not shown). Three clones positive for TgPhIL1 by immunofluorescence were isolated and analyzed by Western blot with the anti-TgPhIL1 antibody. Each of the complemented clones showed levels of TgPhIL1 expression comparable to that of wild-type parasites (Fig. 1C). Two clones, RH*ΔTgPhIL1/PhIL1-C5* and RH*ΔTgPhIL1/PhIL1-C7*, were chosen arbitrarily for subsequent experiments.

### Morphology of the *TgPhIL1* knockout parasites

**Figure 2:**
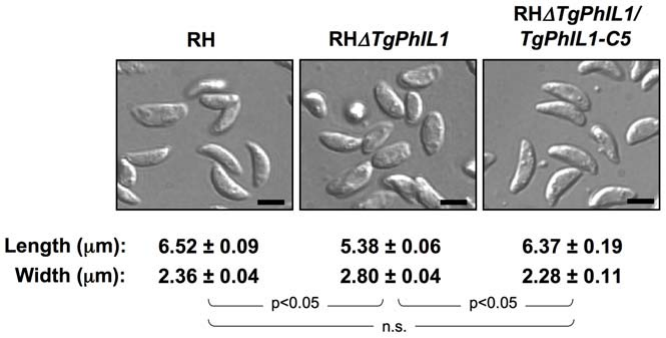
Parasites lacking *TgPhIL1* are shorter and wider than parasites that express *TgPhIL1*. The maximum length and width of individual wild-type (RH), RH*ΔTgPhIL1,* and RH*ΔTgPhIL1/PhIL1-C5* parasites were measured in differential interference contrast images such as those shown. The experiment was done in triplicate, and numbers indicate the average measurements from 100 parasites/experiment, in µm, plus or minus standard error. The RH*ΔTgPhIL1* parasites are shorter and wider than either the wild-type or complemented clones (unpaired student's t-test, p<0.05; n.s.  =  not significant). Scale bars  = 5 µm.

In the course of generating the RH*ΔTgPhIL1* parasites, it was obvious by light microscopy that these parasites had an altered morphology compared to wild-type (RH) parasites (Fig. 2, compare left and middle panels). To quantify this difference, parasites were measured along their long and short axes at their widest point, confirming that the RH*ΔTgPhIL1* parasites are indeed shorter and wider than the RH parasites from which they were derived (Fig. 2). Importantly, the shape of the complemented clones appeared similar to that of wild-type parasites, and morphometric analysis confirmed that complementation of *TgPhIL1* had indeed restored the length and width of the mutant to wild-type values (Fig. 2, compare left and right panels).

To determine whether the morphological effects of the knockout are further accentuated under conditions of osmotic stress, the parasites were treated with various concentrations of HgCl_2_, which disrupts water balance across the plasma membrane by inhibition of aquaporin channels [31], [32]. Both the RH*ΔTgPhIL1* and wild-type parasites swell following treatment with 0.5 µM HgCl_2_, resulting in each case in a decrease in the length to width ratio of ∼15% (Fig. S1). Thus, the already squat-shaped RH*ΔTgPhIL1* parasites become even shorter and wider under conditions of osmotic stress.

### Ultrastructure of the *TgPhIL1* knockout parasites

**Figure 3:**
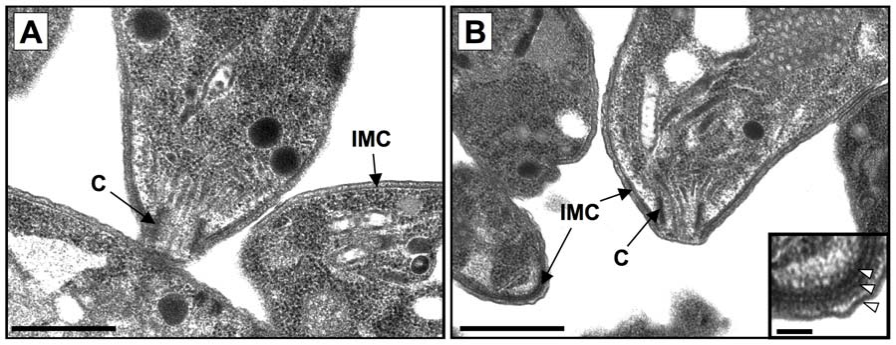
Transmission electron microscopy of *TgPhIL1* knockout parasites. The ultrastructure of the conoid (C), IMC, and spacing between the IMC and plasma membrane is indistinguishable in (**A**) wild-type (RH) and (**B**) RH*ΔTgPhIL1* parasites. Scale bars  = 1 µm. Inset shows an enlarged portion of the RH*ΔTgPhIL1*parasite from the lower left corner of panel B; the normal trilaminar structure of the pellicle is evident (arrowheads). Scale bar  = 0.1 µm.

Given their striking difference in shape, we examined the RH*ΔTgPhIL1* parasites by transmission electron microscopy for ultrastructural changes, particularly in the IMC and/or the region of the pellicle just posterior to the conoid, where TgPhIL1 is known to be concentrated ([18]; see also Fig. S3B). No morphological differences in the IMC, the conoid, or the spacing between the IMC and the plasma membrane were observed (Fig. 3).

### Conoid extension, motility and invasion of the *TgPhIL1* knockout parasites

Since TgPhIL1 localizes in part to a ring just posterior to the conoid, its absence might have an effect on conoid extension. To test this hypothesis, wild-type and RH*ΔTgPhIL1* parasites were treated with 1 µM ionomycin, which induces conoid extension [30]. In response to ionomycin treatment, 86±6.3% of wild-type parasites extended their conoid compared to 14±0.35% of untreated parasites. Similarly, 83±1.8% of the RH*ΔTgPhIL1* parasites extended their conoids in response to ionomycin treatment, compared to 17±2.5% of untreated parasites (Fig. S2), demonstrating that PhIL1 does not play a significant role in conoid extension.

**Figure 4:**
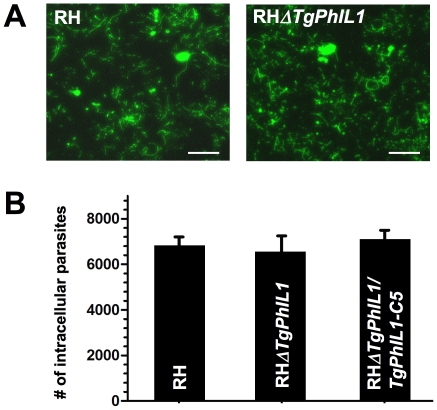
Motility and invasion of the *TgPhIL1* knockout parasites. (A) Motility assays were performed on wild-type (RH) and RH*ΔTgPhIL1* parasites. The density and shape of the trails deposited by RH*ΔTgPhIL1* parasites are qualitatively indistinguishable from those deposited by RH parasites. Scale bars  = 10 µm. (B) Laser-scanning cytometry (LSC)-based invasion assays were performed on wild-type (RH), RH*ΔTgPhIL1*, and RH*ΔTgPhIL1/PhIL1-C5* parasites. Samples were analyzed in duplicate and the results shown are the average from two independent experiments, plus or minus standard error. No significant difference was seen in the ability of the RH*ΔTgPhIL1* parasites to invade host cells when compared to either RH or RH*ΔTgPhIL1/TgPhIL1-C5* parasites (unpaired student's t-test, p>0.05).

Since the shape of *T. gondii* is thought to be important for gliding motility [33], [34], the alteration in morphology caused by *PhIL1* disruption might be expected to affect parasite motility. However, analysis of trails deposited by gliding parasites revealed that the RH*ΔTgPhIL1* parasites were able to undergo both circular and helical gliding, and the number and shape of trails deposited by the knockout parasites was qualitatively indistinguishable from the number and shape deposited by wild-type parasites (Fig. 4A).

To determine whether RH*ΔTgPhIL1* parasites were defective in their ability to invade host cells, quantitative LSC-based invasion assays [29] were performed. Wild-type, RH*ΔTgPhIL1,* and RH*ΔTgPhIL1/PhIL1-C5* parasites were again indistinguishable in their ability to invade host cells (Fig. 4B). Taken together, these data indicate that PhIL1 plays no detectable role in conoid extension, motility, or invasion.

### Other IMC-associated antigens

To determine whether the absence of TgPhIL1 affects the subpellicular microtubules, RH*ΔTgPhIL1* parasites were stained with anti-tubulin and visualized by immunofluorescence microscopy. As shown in Fig. S3A (panel a), the subpellicular microtubules in RH*ΔTgPhIL1* parasites are of similar length and show a similar distribution to those of wild-type parasites. Similarly, no differences were observed in the distribution of: TgIMC1, a major component of the subpellicular network (Fig. S3A, panel b); TgMORN1, which localizes in part to ring-shaped structures associated with the growing ends of the IMC cisternae during daughter cell formation ([26]; Fig. S3A, panel c); and ISP1, a protein associated with the most apical plate of the IMC ([25]; Figs. S3A, panel d). These data, together with the ultrastructural analyses, suggest the IMC, subpellicular network, and microtubules are not significantly altered in the knockout parasites.

### Growth competition assays

**Figure 5:**
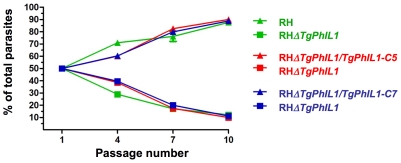
Growth competition of *TgPhIL1* knockout parasites in culture. Equal numbers of parasites from the different lines were mixed pairwise and serially passaged through monolayers of HFF cells. After every three passages, immunofluorescence was performed and 500 parasites were counted and scored as either positive or negative for TgPhIL1. Each color (red, green, and blue) indicates a pair of parasite lines passaged together. Wild-type (RH) parasites (green triangles) and each of the complemented clones (RH*ΔTgPhIL1/TgPhIL1-C5* [red triangles] and RH*ΔTgPhIL1/TgPhIL1-C7* [blue triangles] outgrew the RH*ΔTgPhIL1* parasites (squares) at all time points (passages 4, 7 and 10). Each experiment was performed in duplicate, and the results shown are the average of the three experiments, plus or minus standard error. The difference between the growth of each of the pairs was significant at all passage numbers >1 (unpaired student's t-test, p<0.05).

To assess how well the knockout parasites grow *in vitro,* wild-type and RH*ΔTgPhIL1* parasites were added to confluent monolayers of HFF cells and the number of parasites per vacuole was counted at various times (0–36 hours) post infection. No difference in growth rate between the wild-type and knockout parasites was detected (data not shown). To look for a more subtle growth defect over a longer time period, equal numbers of wild-type and RH*ΔTgPhIL1* parasites were mixed and used to infect the same flask of HFF cells. Each time the parasites lysed the host cell monolayer, culture supernatant containing the released parasites was added to a fresh host cell monolayer. After every third passage, anti-TgPhIL1 immunofluorescence was performed on the released parasites to determine the relative number of wild-type and knockout parasites present. As shown in Fig. 5, wild-type parasites start to outgrow the RH*ΔTgPhIL1* parasites by passage 4 and continue to do so at passages 7 and 10. This same experiment was performed with pairwise cultures of the knockout parasites and each of the complemented lines (*i.e.*, RH*ΔTgPhIL1 vs.* RH*ΔTgPhIL1/TgPhIL1-C5* and RH*ΔTgPhIL1 vs.* RH*ΔTgPhIL1/TgPhIL1-C7*); like the wild-type parasites, the complemented lines outgrow the *PhIL1* knockout parasites at all time points (Fig. 5).

### Decreased survival of *TgPhIL1* knockout parasites *in vivo*

**Figure 6:**
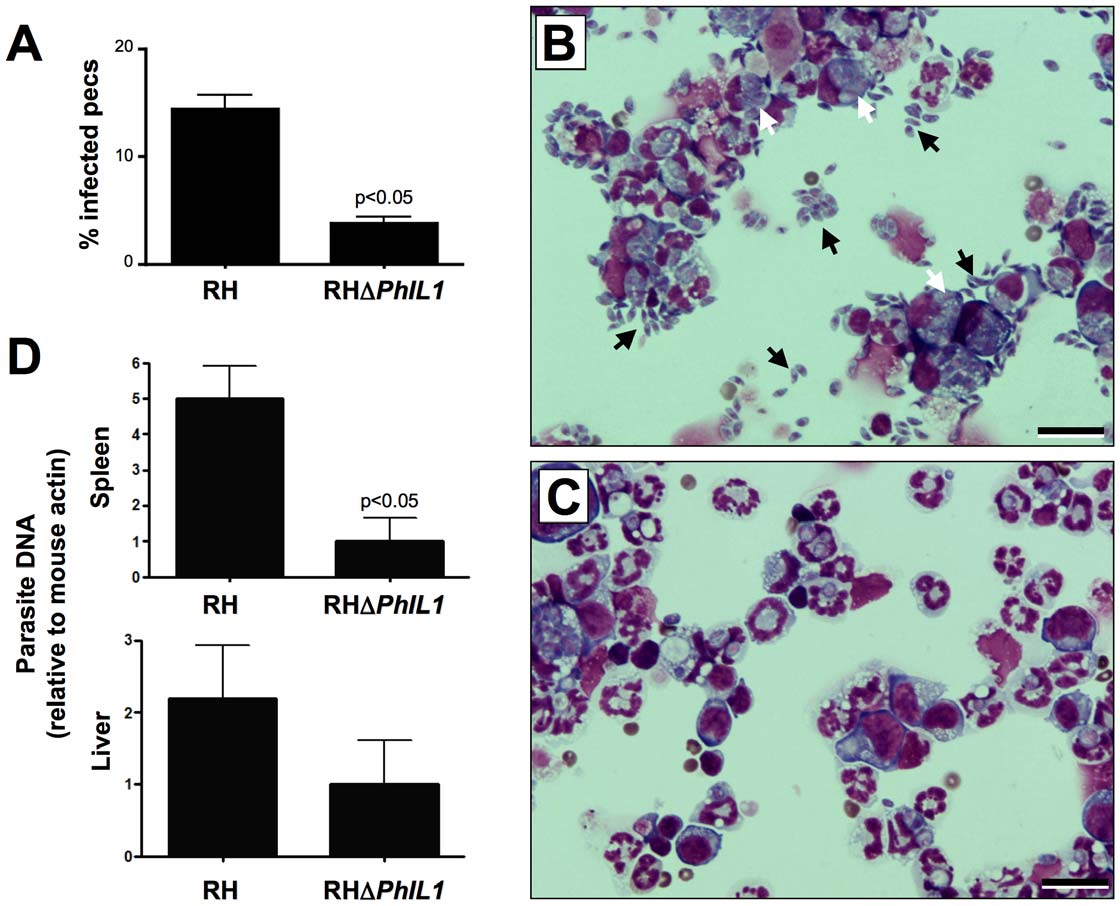
Survival and dissemination of *TgPhIL1* knockout parasite in a mouse model of infection. Mice were infected intraperitoneally with 1×10^4^ wild-type (RH) or RH*ΔTgPhIL1* tachyzoites. (**A-C**) Peritoneal exudate cells (PECs) were harvested seven days post-infection, spun onto coverslips and examined for the presence of parasites. Panel **A** shows that PECs from RH-infected mice carry a significantly higher parasite load than those from RH*ΔTgPhIL1*-infected mice (unpaired student t-test, p<0.05). Results shown are the average from five mice, plus or minus standard error. Panels **B** and **C** show representative examples of the coverslips from which the data in panel **A** were derived (Panel **B**, RH-infected PECs; Panel **C**, RH*ΔTgPhIL1*-infected PECs). Black arrows indicate extracellular parasites and white arrows intracellular parasites. Scale bars  = 20 µm. (**D**) DNA was harvested from spleen and liver samples seven days post-infection, and qPCR was performed to determine the amount of parasite DNA present. Results shown are the average parasite loads from five mice, plus or minus standard error. The liver and spleen each contained a higher load of RH than RH*ΔTgPhIL1* parasites, although only the differences observed in the spleen were statistically significant (unpaired student t-test, p<0.05).

Since the RH*ΔTgPhIL1* parasites were outgrown by wild-type parasites *in vitro*, we tested for a growth difference *in vivo.* Mice were infected intraperitoneally with 1×10^4^ wild-type or RH*ΔTgPhIL1* tachyzoites. Seven days post-infection, large numbers of extracellular and intracellular wild-type parasites were observed in the peritoneal fluid. Significantly fewer free and intracellular parasites were detected in the peritoneal fluid of mice infected with the RH*ΔTgPhIL1* parasites (Fig. 6A-C). The spleen and liver were also harvested seven days after infection, and quantitative PCR (qPCR) was performed to determine the amount of parasite DNA present. As shown in Fig. 6D, less parasite DNA was present in the spleen and liver of mice infected with RH*ΔTgPhIL1* parasites compared to wild-type parasites; this difference was particularly evident in the spleen, where five-fold less RH*ΔTgPhIL1* parasite DNA was observed. These data demonstrate that the *PhIL1* knockout parasites exhibit a reduced ability to survive and/or disseminate in mice when compared to wild-type parasites.

## Discussion

TgPhIL1, which was originally discovered in photolabeling experiments designed to identify novel proteins of the *T. gondii* pellicle [18], is conserved among members of the Phylum Apicomplexa but exhibits very little sequence similarity to non-apicomplexan proteins currently in the sequence databases. Formulating a hypothesis on the function of a protein that contains no identifiable protein domains or homologs of known function is challenging. Based on its localization to the IMC and properties suggestive of both a cytoskeletal and membrane-associated protein, TgPhIL1 could serve to organize or physically connect elements of the parasite cytoskeleton with the membranes of the IMC [18].

As a first step in elucidating the function of TgPhIL1, we generated a *TgPhIL1* knockout parasite line. The knockout parasites have a conspicuously altered morphology, being shorter and wider than the wild-type parasites from which they were derived. Given that the shape of *T*. *gondii* is thought to be important for motility [33], [34], it was surprising that the RH*ΔTgPhIL1* parasites were capable of normal motility *in vitro*, although we cannot rule out some more subtle effect on *in vitro* motility or a defect that would only be apparent in an infected organism, where the parasites are required to travel longer distances and through various biological barriers [35]. Because PhIL1 localizes to a ring-like structure at the posterior end of the conoid, we also hypothesized that the protein might play some role in conoid extension; however, conoid extension was unaffected by disruption of *TgPhIL1*.

Although indistinguishable from wild-type parasites in a number of respects, the knockout parasites showed significantly slower growth in culture. The growth defect observed in culture translated into a significant decrease in parasite fitness in a mouse model of infection. The number of *RHΔTgPhIL1* parasites detected in the liver, spleen and peritoneal fluid of infected mice was consistently lower with *RHΔTgPhIL1* parasites than with wild-type parasites. No difference was observed in the survival of mice infected with wild-type and *RHΔTgPhIL1* parasites, with all mice dying around day 8 (data not shown). However, these experiments were done with a single dose of 1×10^4^ parasites; a more extensive analysis, using a range of parasite inocula, would be required to establish whether the decreased fitness of the knockout parasites affects host survival.

An alternative approach to identifying the biological function of TgPhIL1 would be to identify proteins with which it interacts. However, TgPhIL1's highly insoluble nature [18] makes coimmunoprecipitation studies to identify interacting proteins technically challenging. In *P. falciparum,* a genome-wide yeast-two-hybrid screen has been performed, with the results available online (www.plasmodb.org). In this screen, the *P. falciparum* homologue of TgPhIL1 (PFA0440w) was shown to interact with another *P. falciparum* protein (PFF0325c), and a homolog of this *P. falciparum* PhIL1-binding protein exists in *T. gondii* (38.m01070). While PFF0325c shows no homology to non-apicomplexan proteins, it was found to interact in the yeast-two-hybrid screen with five proteins in addition to PfPhIL1 (PFA0110w, PFA0285c, PF10_0378, PF08_0137, PFD0320c). Two of these are DnaJ-domain containing proteins, one is predicted to be exported, and several have homologs in *T. gondii.* As additional functional annotation of genes from other organisms becomes available, new insights into the function of the potential PhIL1-binding proteins and of PhIL1 itself may become apparent.

If one of the primary functions of TgPhIL1 is to provide structural stability to the parasite, as suggested by the data reported here, a reduced ability of the knockout parasites to tolerate osmotic or mechanical stresses encountered during *in vivo* infection might be an underlying cause of the parasite's reduced fitness. Intriguingly, disruption of the IMC-associated proteins IMC1a and IMC1b in *Plasmodium berghei* also alters the shape and reduces the mechanical stability of sporozoites and ookinetes, respectively [36], [37]. The rate of parasite proliferation early during infection may affect parasite loads later, as the parasite has a limited time window in which to proliferate before the immune system becomes fully activated. The development of drugs that target components of the IMC and cytoskeleton may therefore be a potentially useful approach to disease management.

In the context of a population of parasites, even relatively subtle growth defects such as the one reported here are likely to present a significant selective disadvantage over time, since parasites exhibiting such a defect will be outcompeted during infection by parasites whose growth is not impaired. Several other *T. gondii* proteins have recently been identified that are non-essential, but make a clear contribution to parasite growth and fitness (*e.g.,* [38]–[40] and GEW, unpublished data). With the recent development of highly efficient methods for gene replacement in *T. gondii* [41], [42], many more such genes are likely to be identified. In these cases, it may be that we simply do not know the relevant assay, host, or environmental conditions to reveal a clear phenotype when the gene is disrupted. The overall fitness of a given parasite strain will be determined by both its essential genes and the additive effect of genes which may not be essential but provide some degree of fitness benefit to the parasite in the context of particular host organisms or environmental conditions. A broader experimental context is likely to be required for elucidating the biological function of genes such as TgPhIL1, which are beneficial to the parasite but not strictly essential.

## Supporting Information

Figure S1 **Osmotic stress causes an equivalent amount of swelling in RH and** ***TgPhIL1*** **knockout parasites.** Wild-type (RH) and RH*ΔTgPhIL1* parasites were incubated for 15 minutes in the presence or absence of varying concentrations of HgCl_2_. The maximum length and width of individual parasites, in µm, was determined as described in Materials and Methods and expressed as the length to width ratio (LW ratio). The experiment was done in duplicate, and numbers indicate the average measurements from 100 parasites/experiment plus or minus standard error. Statistical significance was calculated using an unpaired student's t-test.
(TIF)

Figure S2 **Conoid extension in RH and** ***TgPhIL1*** **knockout parasites.** Wild-type (RH) and RH*ΔTgPhIL1* parasites were incubated for 5 minutes in the presence (+) or absence (-) of 1 µM ionomycin, and the percentage of parasites with extended conoids was scored by phase microscopy. The results shown are the average of two experiments +/- standard error. The two parasite lines showed no significant difference in ionomycin-induced conoid extension (unpaired student's t-test, p>0.05).
(TIF)

Figure S3 **Localization of α-tubulin, TgIMC1, TgMORN1 and ISP1 in** ***TgPhIL1*** **knockout parasites.** (**A**) The distributions of α-tubulin, TgIMC1, TgMORN1 and ISP1 were examined in wild-type (RH) and RH*ΔTgPhIL1* parasites by immunofluorescence microscopy. (**a**) The splayed subpellicular microtubules of the parasite are indistinguishable in the wild-type and RH*ΔTgPhIL1* parasites. (**b**) TgIMC1 localizes to the periphery of the mother cell and the growing daughters during endodyogeny in both parasite lines. (**c**) TgMORN1 localizes to the basal end of the parasite, the centrocone, and a pair of rings around the dividing nucleus during endodyogeny in both parasite lines (red  =  TgMORN1; green  =  TgSAG1, a plasma membrane marker). (**d**) ISP1 is found in an indistinguishable apical cap-like distribution in the two parasite lines (red  =  ISP1; green  =  polyclonal serum directed against total tachyzoite antigens). Scale bars  = 5 µm. (**B**) The localization of PhIL1 is shown for reference: as previously described [18], PhIL1-YFP localizes to the parasite periphery and is concentrated at both the basal end and the apical end just posterior to the conoid (YFP fluorescence, middle panel). The corresponding DIC and merged fluorescence/DIC images are shown in the upper and lower panels, respectively. Scale bar  = 5 µm.
(TIF)
