# Supplementary figures and images for: Targeted Disruption of TgPhIL1 in Toxoplasma gondii Results in Altered Parasite Morphology and Fitness
Source: PLoS One. 2011 Aug 25;6(8):e23977. doi: 10.1371/journal.pone.0023977 (PMC3162014; doi:10.1371/journal.pone.0023977)

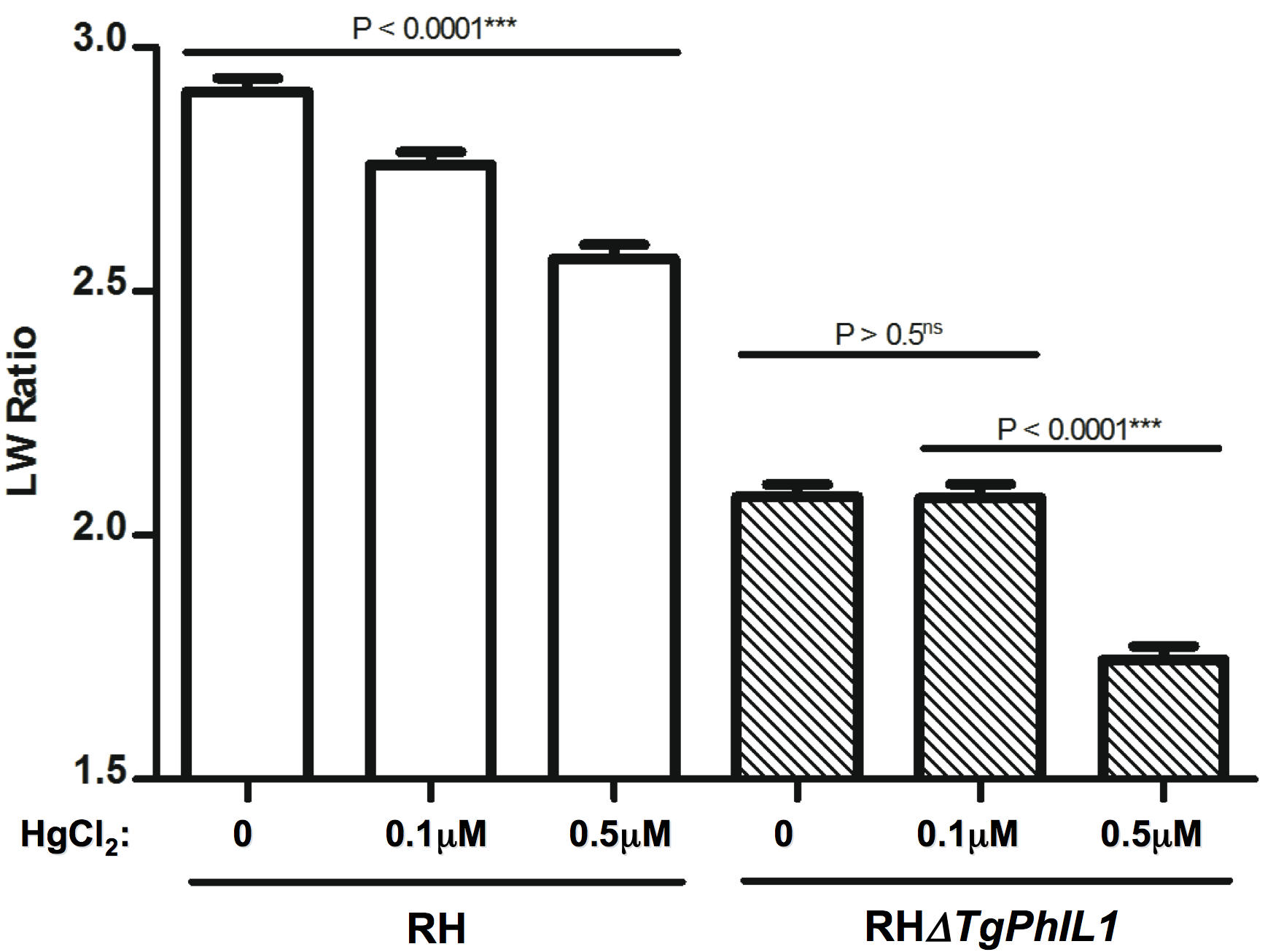

Supplement: Figure S1 — Osmotic stress causes an equivalent amount of swelling in RH and TgPhIL1 knockout parasites. Wild-type (RH) and RHΔTgPhIL1 parasites were incubated for 15 minutes in the presence or absence of varying concentrations of HgCl2. The maximum length and width of individual parasites, in µm, was determined as described in Materials and Methods and expressed as the length to width ratio (LW ratio). The experiment was done in duplicate, and numbers indicate the average measurements from 100 parasites/experiment plus or minus standard error. Statistical significance was calculated using an unpaired student's t-test. (TIF) [file pone.0023977.s001.tif]

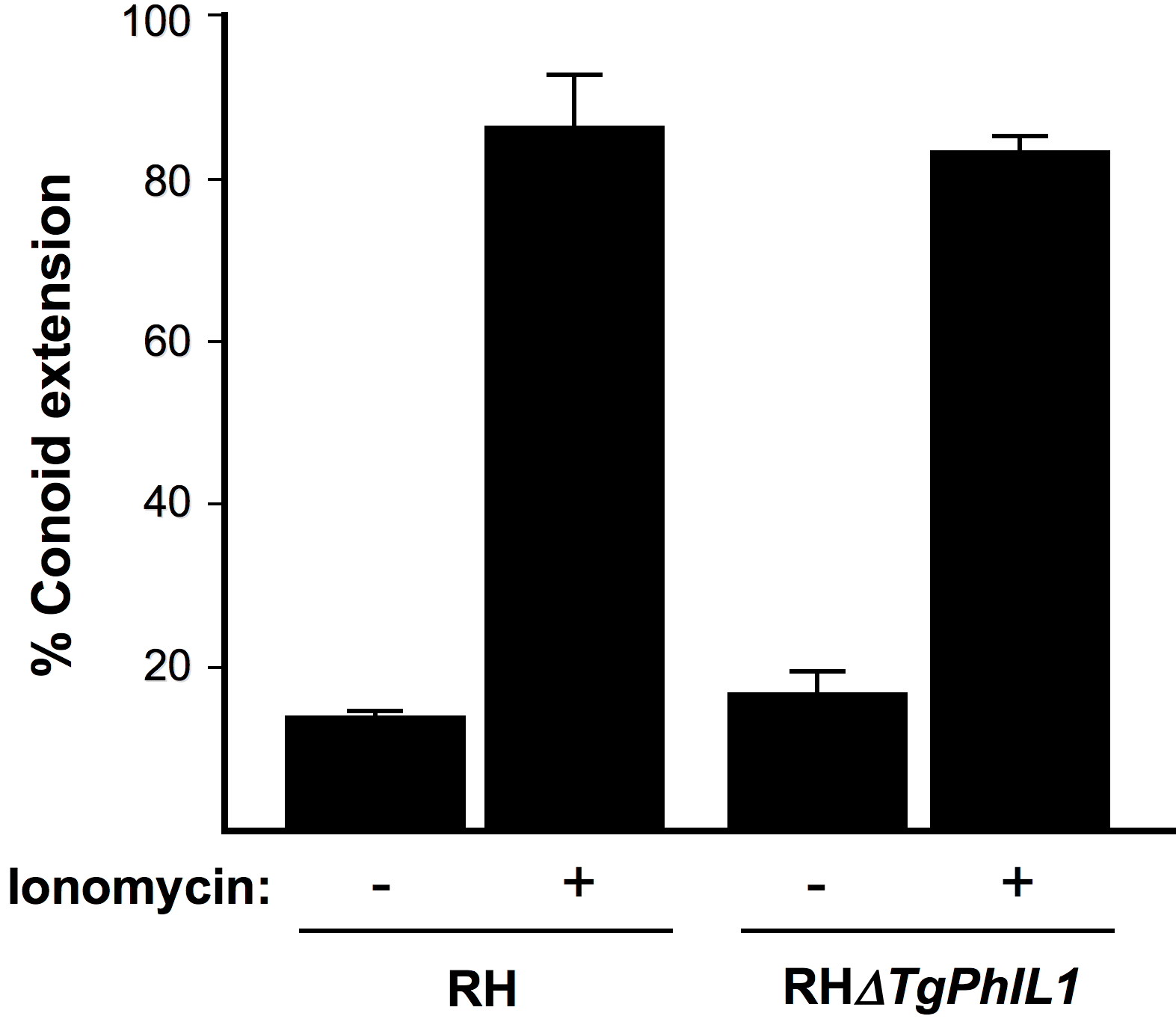

Supplement: Figure S2 — Conoid extension in RH and TgPhIL1 knockout parasites. Wild-type (RH) and RHΔTgPhIL1 parasites were incubated for 5 minutes in the presence (+) or absence (-) of 1 µM ionomycin, and the percentage of parasites with extended conoids was scored by phase microscopy. The results shown are the average of two experiments +/- standard error. The two parasite lines showed no significant difference in ionomycin-induced conoid extension (unpaired student's t-test, p>0.05). (TIF) [file pone.0023977.s002.tif]

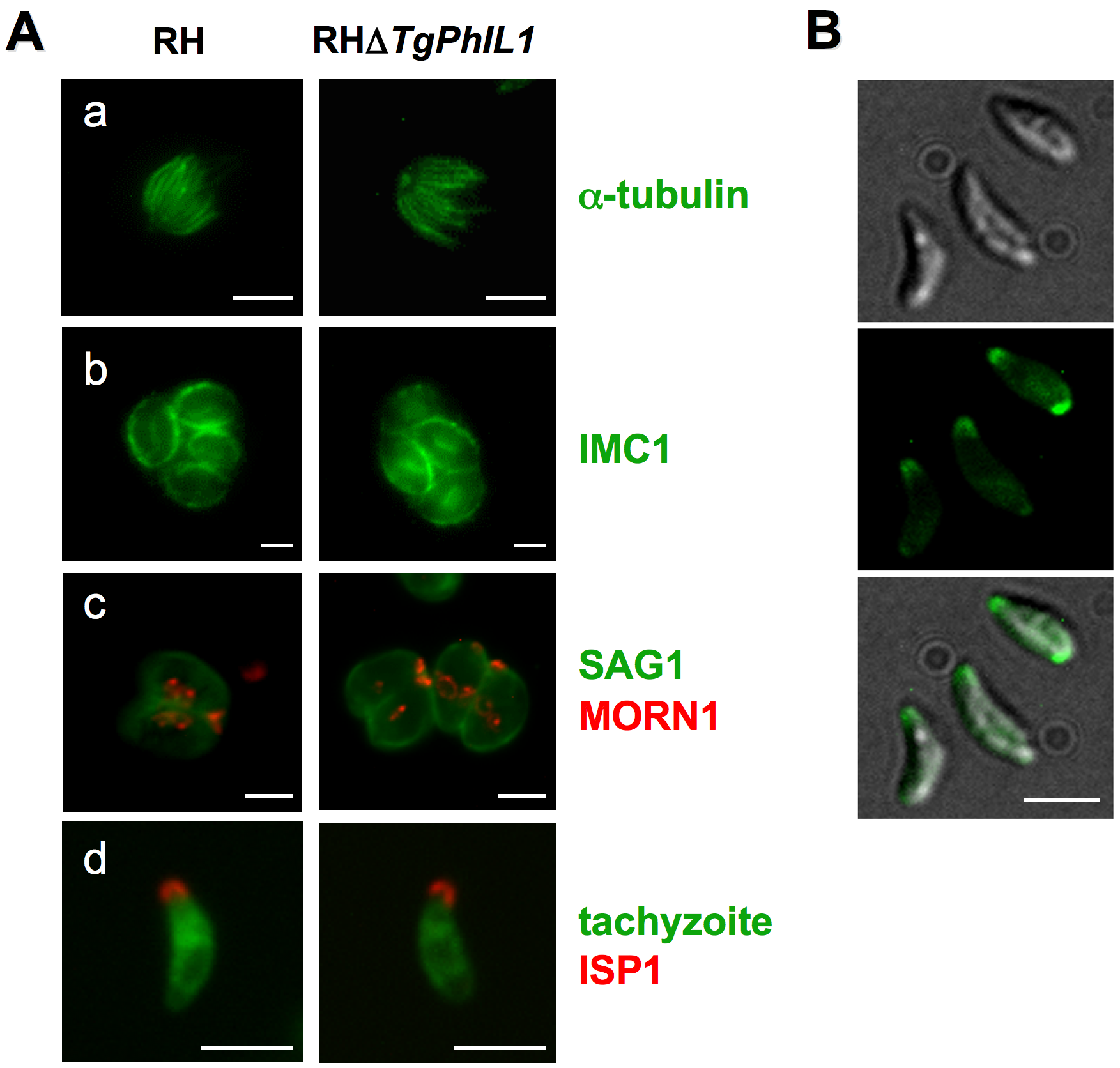

Supplement: Figure S3 — Localization of α-tubulin, TgIMC1, TgMORN1 and ISP1 in TgPhIL1 knockout parasites. (A) The distributions of α-tubulin, TgIMC1, TgMORN1 and ISP1 were examined in wild-type (RH) and RHΔTgPhIL1 parasites by immunofluorescence microscopy. (a) The splayed subpellicular microtubules of the parasite are indistinguishable in the wild-type and RHΔTgPhIL1 parasites. (b) TgIMC1 localizes to the periphery of the mother cell and the growing daughters during endodyogeny in both parasite lines. (c) TgMORN1 localizes to the basal end of the parasite, the centrocone, and a pair of rings around the dividing nucleus during endodyogeny in both parasite lines (red = TgMORN1; green = TgSAG1, a plasma membrane marker). (d) ISP1 is found in an indistinguishable apical cap-like distribution in the two parasite lines (red = ISP1; green = polyclonal serum directed against total tachyzoite antigens). Scale bars = 5 µm. (B) The localization of PhIL1 is shown for reference: as previously described [18], PhIL1-YFP localizes to the parasite periphery and is concentrated at both the basal end and the apical end just posterior to the conoid (YFP fluorescence, middle panel). The corresponding DIC and merged fluorescence/DIC images are shown in the upper and lower panels, respectively. Scale bar = 5 µm. (TIF) [file pone.0023977.s003.tif]
